# Disseminated and fatal infection by *Schizophyllum commune* in a harbor seal (*Phoca vitulina*) kept under human care in Brazil

**DOI:** 10.1007/s11259-026-11559-9

**Published:** 2026-10-07

**Authors:** Jhonatan Henrique Lima da Rocha, Laura Chrispim Reisfeld, Paloma Canedo, Islene Silva Santos, Jordana Barros, Jessica de Brito Ferreira Nascimento, Isabella Galesi Tallach, Ketlyn Bolsachini Figueiredo, Ana Carolina Souza Ramos de Carvalho, Lidia Midori Kimura, Leonardo José Tadeu de Araújo, Juliana Possatto Fernandes Takahashi, Juliana Fernandes de Paula Castro, Pedro Enrique Navaz Suarez, Jamile Macedo Garcia, Cinthya dos Santos Cirqueira Borges, Natália Coelho Couto de Azevedo Fernandes, Juliana Mariotti Guerra

**Affiliations:** 1 Laboratório de Patologia Comparada de Animais Selvagens, Departamento de Patologia, Faculdade de Medicina Veterinária e Zootecnia, Universidade de São Paulo, Av. Prof. Dr. Orlando Marques de Paiva, 87 Cidade Universitária “Armando Salles de Oliveira”, São Paulo, 05508-270 SP Brasil; 2 Aquário de São Paulo, São Paulo, Brasil; 3 Núcleo de Patologia Quantitativa, Centro de Patologia, Centro de Patologia, Instituto Adolfo Lutz, São Paulo, Brasil; 4 Programa de Pós-graduação da Coordenadoria de Controle de Doenças da Secretaria do Estado da Saúde do Estado de São Paulo, São Paulo, Brasil; 5 Laboratório Zooscience, São Paulo, Brasil; 6 Núcleo de Hematologia e Bioquímica, Centro de Patologia, Centro de Patologia, Instituto Adolfo Lutz, São Paulo, Brasil; 7 Núcleo de Anatomia Patológica, Centro de Patologia, Instituto Adolfo Lutz, Centro de Patologia, São Paulo, Brasil

**Keywords:** *Schizophyllum*, *Phoca vitulina*, Basidiomycete, Encephalitis, Pneumonia, Molecular diagnosis, Wildlife pathology, Marine mammal

## Abstract

**Background:**

*Schizophyllum commune* is an environmental basidiomycete increasingly recognized as an opportunistic pathogen in humans and domestic animals, whereas infections in wildlife remain rarely reported. In marine mammals, fungal infections involving the central nervous system and respiratory tract are uncommon and may pose diagnostic challenges.

**Case report:**

A captive 5-year-old female harbor seal (*Phoca vitulina*) from Brazil developed progressive neurological abnormalities over approximately 11 months, initially characterized by cervical dystonia and head deviation, followed by respiratory distress, tremors, opisthotonos, hyperthermia, and apnea. Despite supportive treatment, the animal died. Histopathological examination revealed severe pyogranulomatous encephalitis involving the cerebral cortex and severe multifocal to coalescing pyogranulomatous pneumonia, both associated with extensive necrosis and numerous intralesional hyaline, pauciseptate fungal hyphae measuring approximately 2–4 μm in diameter, with irregular branching at angles ranging from 30° to 90°, highlighted by Grocott’s methenamine silver staining. Molecular investigation was performed using panfungal PCR targeting the D1/D2 domain of the 28 S rDNA and the internal transcribed spacer (ITS) region. Sequence analysis, particularly of the ITS region, identified the fungus as *S. commune*, with phylogenetic analysis further supporting its placement within the *S. commune* clade.

**Conclusion:**

To the authors’ knowledge, this is the first report of disseminated *S. commune* infection involving the central nervous system and lungs in a harbor seal. The case highlights *S. commune*’s potential to cause severe systemic disease in wildlife and emphasizes the value of integrating histopathological examination with molecular and phylogenetic analyses for accurate identification of fungal pathogens.

**Supplementary Information:**

The online version contains supplementary material available at https://doi.org/10.1007/s11259-026-11559-9.

## Introduction

*Schizophyllum* (Schizophyllaceae, Agaricales) is a ubiquitous environmental basidiomycete commonly associated with decaying organic material, particularly rotting wood and plant debris (Mahajan 2022). Although traditionally regarded as a saprophyte, *S. commune* is increasingly recognized as an emerging opportunistic pathogen, mainly associated with allergic and invasive respiratory diseases in humans, including sinusitis, allergic bronchopulmonary mycosis, and mycotic bronchopneumonia (Chowdhary et al. 2013).

Reports of *S. commune* infection in animals remain exceedingly rare. Naturally occurring infections have been documented in dogs (*Canis lupus familiaris*) (Kano et al. 2002; Tanaka et al. 2008; Mori et al. 2009; Hakamata et al. 2019; Go et al. 2022), cheetahs (*Acinonyx jubatus*) (Yoshizawa et al. 2017; Noguchi et al. 2023), and harbor seals (*Phoca vitulina*) (Hanafusa et al. 2016; Fujii et al. 2024), affecting both immunocompetent and immunocompromised hosts. Although most infections are associated with the respiratory tract, some hosts may develop systemic manifestations, with involvement of the skin, eyes, bones, and, occasionally, the central nervous system, one of the most frequently affected extrapulmonary sites (Tanaka et al. 2008; Yoshizawa et al. 2017; Nagashima et al. 2024).

Identification of *Schizophyllum commune* is challenging due to the limited reliability of morphological characterization in culture. Diagnostic structures such as basidiocarps, dikaryotic hyphae, and clamp connections are inconsistently observed, and their morphology may closely resemble that of other filamentous fungi. As a result, accurate species identification typically depends on molecular methods, particularly ribosomal DNA sequencing (Sigler et al. 1995; Won et al. 2012).

Despite the increasing recognition of *S. commune* as a pathogen, its ecoepidemiology remains incompletely understood. Most human cases have been reported in Asia, particularly in Japan, suggesting possible geographic clustering or underdiagnosis in other regions (Xue et al. 2025). In contrast, reports in animals, particularly in wildlife species, remain limited.

In this context, the present study aimed to describe the clinical presentation, histopathological findings, and molecular identification of a disseminated and fatal *S. commune* infection in a harbor seal (*Phoca vitulina*) maintained under human care in Brazil, thereby contributing novel data on *S. commune* infection in marine mammals and expanding current understanding of its pathogenic potential in non-human hosts, and extending its known geographic distribution.

## Case report

A 5-year-old female harbor seal (*Phoca vitulina*), born *ex situ* at a zoological institution in Belgium, was transferred to the São Paulo Aquarium in Brazil in 2018 at approximately one year of age. The seal remained clinically stable until February 2022, when she developed mild left-sided cervical dystonia. Radiographic examination of the skull, cervical spine, and thoracic limbs revealed no significant abnormalities. Treatment with carprofen (2 mg/kg) and laser therapy was initiated. However, the clinical condition did not improve after one week of treatment. Additional radiographic examinations during follow-up revealed no abnormalities. Hematologic and serum biochemical analyses revealed leukocytosis with neutrophilia and eosinophilia, hyperfibrinogenemia, increased blood urea nitrogen (BUN), and elevated serum activities of gamma-glutamyl transferase (GGT) and creatine kinase (CK) (Supplementary Table 1). A molecular diagnostic panel targeting multiple infectious pathogens in ocular and nasal swab samples, together with serologic testing for *Toxoplasma gondii*, yielded negative results (Supplementary Table 2).

Prednisone (0,5 mg/kg, PO, SID) was administered for 10 days; however, no clinical improvement was observed. Given the persistence and progression of the neurological signs, together with the loss of both superficial and deep nociception, an ischemic neurological event was considered among the differential diagnoses. Treatment with amitriptyline (1 mg/kg PO, SID) was initiated, resulting in a gradual improvement in the animal’s postural abnormalities, and the animal remained clinically stable over the following months.

In January 2023, the animal exhibited progressive clinical deterioration characterized by respiratory signs and anorexia. Hematologic and biochemical analyses revealed mild normocytic normochromic anemia, hypoproteinemia with hypoglobulinemia, and mild hematuria. Supportive treatment, including analgesia, antiemetics, and fluid therapy, resulted in transient clinical improvement, with partial recovery of appetite. Thoracic radiographs and ultrasonography identified bilateral pulmonary nodules. Over the following week, neurological dysfunction progressed with tremors, opisthotonos, hyperthermia, and episodes of apnea. Repeat hematologic and biochemical analyses demonstrated leukocytosis with neutrophilia and eosinophilia, thrombocytosis, hyperfibrinogenemia, azotemia, elevated hepatic enzyme, hyperlactatemia, and persistent hematuria. Despite intensive supportive care, including midazolam, vitamin E supplementation, fluid therapy, and acupuncture, the animal showed only transient improvement (Supplementary Table 1). The animal developed cardiopulmonary arrest the following day and died despite resuscitative efforts.

**Fig. 1:**
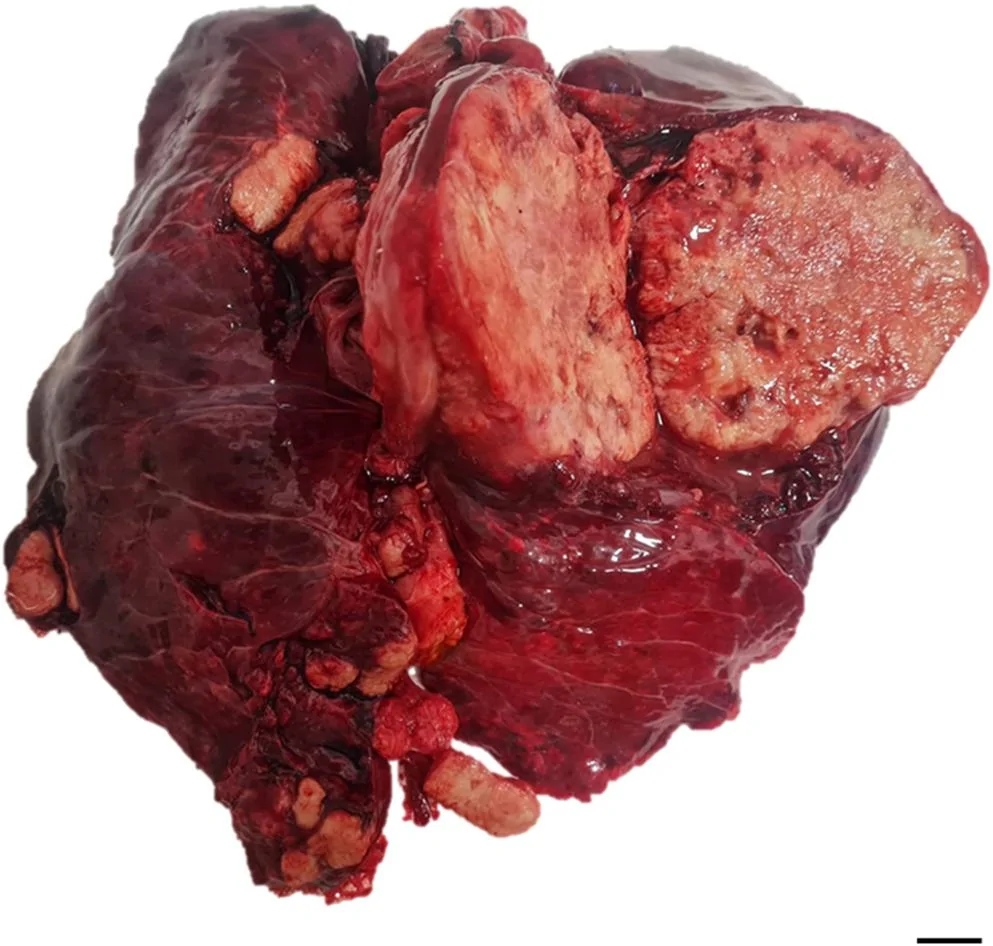
Lungs from a harbor seal (*Phoca vitulina*) with disseminated *Schizophyllum commune* infection. The pulmonary parenchyma is expanded by multiple, multifocal to coalescing, firm, well-demarcated, white to pale tan nodules. A large multilobulated mass, measuring approximately 10 cm in diameter, replaces most of the left middle lung lobe and compresses the adjacent pulmonary parenchyma. On the cut section, the mass is predominantly homogeneous and white to tan, with multifocal cavitary areas measuring approximately 0.5–1.0 cm in diameter that drain abundant, thick, granular, yellow-red material. Bar = 1 cm

At necropsy, the animal was in good body condition. Approximately 600 mL of blood was present within the pleural cavity. Both lungs were diffusely dark red and were expanded by numerous, multifocal to coalescing, well-demarcated, firm, white to pale tan nodules distributed throughout the pulmonary parenchyma (Fig. 1). The largest lesion, located in the left middle lung lobe, consisted of a multilobulated mass measuring approximately 10 cm in diameter that replaced most of the affected lobe and caused marked right dorsolateral displacement of the heart. On the cut section, the mass was solid, homogeneous, white to pale tan, with multifocal areas of hemorrhage and necrosis. The overlying visceral pleura was focally ruptured, extending into the adjacent pulmonary parenchyma. Multiple additional nodules, ranging from approximately 0.5 to 4 cm in diameter, were scattered throughout the remaining pulmonary lobes, several of which protruded from the pleural surface. The liver was diffusely mottled red-yellow. Mild congestion was observed in the spleen, kidneys, mesenteric vasculature, and meningeal vessels. Fecal flotation was negative for parasitic ova and oocysts. Samples of the brain, lungs, liver, kidneys, spleen, ovary, uterus, heart, stomach, and small intestine were collected, fixed in 10% neutral-buffered formalin, routinely processed, embedded in paraffin, sectioned at 4 μm, and stained with hematoxylin and eosin (HE) for histopathologic evaluation.

**Fig. 2:**
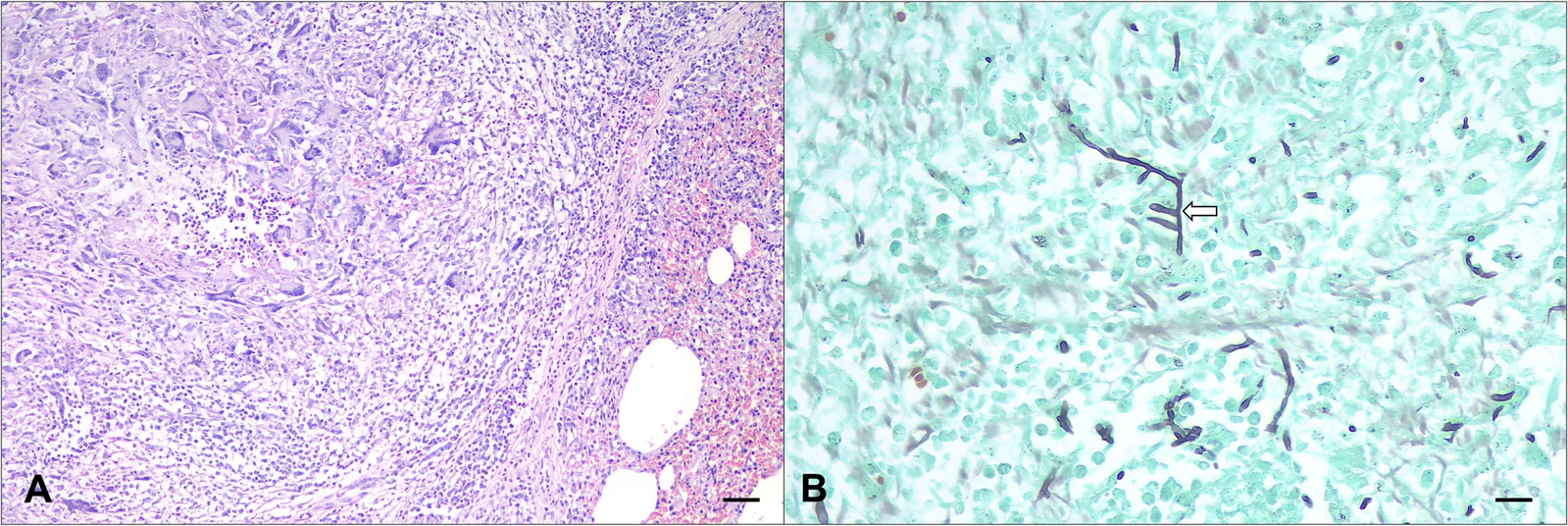
Lung, harbor seal (*Phoca vitulina*) with disseminated *Schizophyllum commune* infection. (**A**) Severe multifocal to coalescing pyogranulomatous pneumonia characterized by extensive necrosis surrounded by macrophages, multinucleated giant cells, neutrophils, and fewer lymphocytes and plasma cells. Hematoxylin and eosin (HE). (**B**) Grocott’s methenamine silver (GMS) stain highlighting numerous intralesional hyaline, pauciseptate fungal hyphae measuring approximately 2–4 μm in diameter with predominantly right-angle branching

**Fig. 3:**
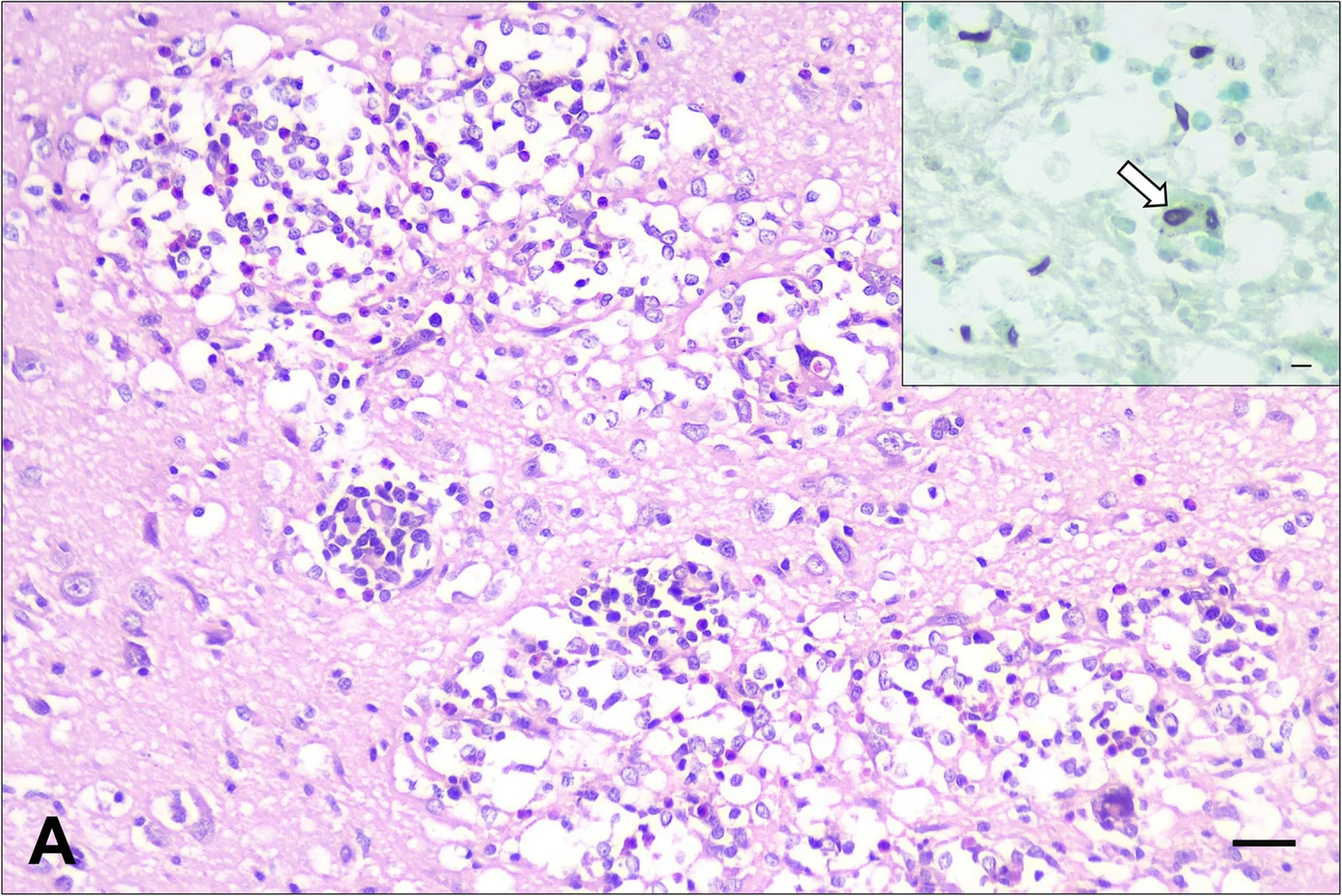
Brain, harbor seal (*Phoca vitulina*) with disseminated *Schizophyllum commune* infection. (**A**) Multifocal histiocytic and lymphoplasmacytic encephalitis with rare multinucleated giant cells. HE. Inset: GMS stain highlighting scarce intralesional hyaline, pauciseptate fungal hyphal fragments (arrow)

Histopathologic examination of the lungs revealed severe multifocal to coalescing pyogranulomatous pneumonia, characterized by extensive areas of necrosis predominantly surrounded by macrophages, multinucleated giant cells, and neutrophils, with fewer lymphocytes and plasma cells (Fig. 2). Numerous intralesional hyaline, pauciseptate fungal hyphae measuring approximately 2–4 μm in diameter, with irregular branching at angles ranging from 30° to 90°, were distributed throughout the lesions. The hyphae were frequently observed within multinucleated giant cells and occasionally within blood vessels. Pulmonary lesions were additionally characterized by moderate interlobular fibrosis, multifocal lymphocytic septal infiltrates, extensive hemorrhage, and adjacent areas of alveolar atelectasis, edema, and congestion. Grocott’s methenamine silver (GMS) stain strongly highlighted abundant intralesional fungal hyphae (Fig. 2). The cerebral cortex revealed focally extensive histiocytic and lymphoplasmacytic encephalitis, with rare Langhans-type multinucleated giant cells (Fig. 3). Similar hyaline, pauciseptate fungal hyphae were identified within the inflammatory lesions, although less abundantly than in the lungs, and were highlighted by GMS staining. Systemic lesions included multifocal centrilobular to midzonal hepatic hemorrhage associated with hepatocellular necrosis and apoptosis, mild-to-moderate multifocal sinusoidal congestion, mild multifocal lymphoplasmacytic portal hepatitis, mild proliferative ductular reaction, multifocal mesothelial reactivity, and occasional intravascular fibrin thrombi. A focally extensive area of acute subcapsular hepatic hemorrhage was also present. Renal lesions consisted of multifocal mild-to-moderate intratubular mineral deposition associated with mild multifocal mononuclear interstitial nephritis, mild vacuolar degeneration of the tunica media of medium-caliber medullary arteries, and multifocal intravascular fibrin thrombi. The spleen exhibited moderate multifocal hemosiderosis and moderate extramedullary hematopoiesis with numerous megakaryocytes. No significant microscopic lesions were identified in the ovaries, uterus, heart, stomach, or small intestine.

For molecular investigations, genomic DNA was extracted from fresh-frozen lung tissue using the Biogene DNA/RNA Extraction Kit (Bioclin, Belo Horizonte, Brazil) according to the manufacturer’s instructions with minor modifications. DNA quality and amplifiability were assessed by real-time PCR targeting the endogenous eukaryotic 18 S rRNA gene as previously described (Rojas et al. 2010) yielding a quantification cycle (Cq) value of 7.11. Samples were subsequently screened for herpesviruses using a pan-herpesvirus nested PCR targeting the DNA polymerase gene (VanDevanter et al. 1996) and a gammaherpesvirus-specific PCR assay (Ehlers et al. 2008), both of which were negative.

Panfungal conventional PCR assays were performed targeting two ribosomal DNA regions. The internal transcribed spacer (ITS) region was amplified using primers ITS3-F (5′-GCATCGATGAAGAACGCAGC-3′) and ITS4-R (5′-TCCTCCGCTTATTGATATGC-3′), while the D1/D2 domain of the 28 S rDNA gene was amplified using primers NL1 (5′-GCATATCAATAAGCGGAGGAAAAG-3′) and NL4 (5′-GGTCCGTGTTTCAAGACGG-3′) (Leaw et al. 2006). PCR reactions were performed in a final volume of 25 µL containing 5 µL of DNA template, 0.5 µL of each primer (20 µM), 12.5 µL of GoTaq^®^ Green Master Mix (Promega, Madison, WI, USA), and nuclease-free water. Thermal cycling conditions included an initial denaturation at 95 °C for 5 min, followed by 40 cycles of 95 °C for 30 s, 55 °C for 60 s, and 72 °C for 30 s, with a final extension at 72 °C for 10 min. Purified genomic DNA from an *Aspergillus* spp. proficiency testing sample (Controllab, Rio de Janeiro, Brazil) was used as a positive control, whereas nuclease-free water served as the negative control. Amplified PCR products were treated with ExoSAP-IT™ PCR Product Cleanup Reagent (Applied Biosystems, Foster City, CA, USA) according to the manufacturer’s instructions and directly sequenced using the ABI BigDye Terminator Cycle Sequencing Kit v3.1 on an ABI Prism 3500 Genetic Analyzer (Applied Biosystems, Foster City, CA, USA). Consensus sequences were assembled using ClustalX version 2.0.8 and manually edited in BioEdit version 7.2. Homology searches were performed against sequences deposited in the GenBank/EMBL/DDBJ databases using BLASTn (National Center for Biotechnology Information, Bethesda, MD, USA).

For phylogenetic analysis, ITS and LSU sequences were aligned separately with homologous reference sequences retrieved from GenBank using MEGA version 12.1.2 (Tamura et al. 2021). Maximum Likelihood trees were inferred using the Kimura two-parameter (K2P) nucleotide substitution model, and node support was evaluated by 1,000 bootstrap replicates.

BLASTn analysis of the D1/D2 domain of the 28 S rDNA amplified with primers NL1 and NL4 (GenBank accession number PZ556955) demonstrated complete nucleotide identity with both *S. radiatum* strain CBS199.27 (LT217570.1) and *S. commune* isolate VXL155 (MW553722.1), each showing 100% nucleotide identity, 100% query coverage, and E-values of 0.0. Additional *S. commune* sequences, including isolates MTP78 (PX211013.1), IFM 65,102 (LC798837.1), IFM 65,470 (LC798857.1), M17 (PX850379.1), strain 736 (OR394622.1), and MUCL30748 (LT217566.1), exhibited 99.83% nucleotide identity. Similarly, multiple *S. radiatum* sequences, including isolate LF00513 (PZ352953.1) and strains UTHSCDI14-8 (LT217577.1) and CBS301.32 (LT217569.1), displayed nucleotide identities ranging from 99.66% to 99.83%. These findings confirmed assignment to the genus *Schizophyllum* but showed that the D1/D2 region lacked sufficient variability for reliable species-level discrimination.

**Fig. 4:**
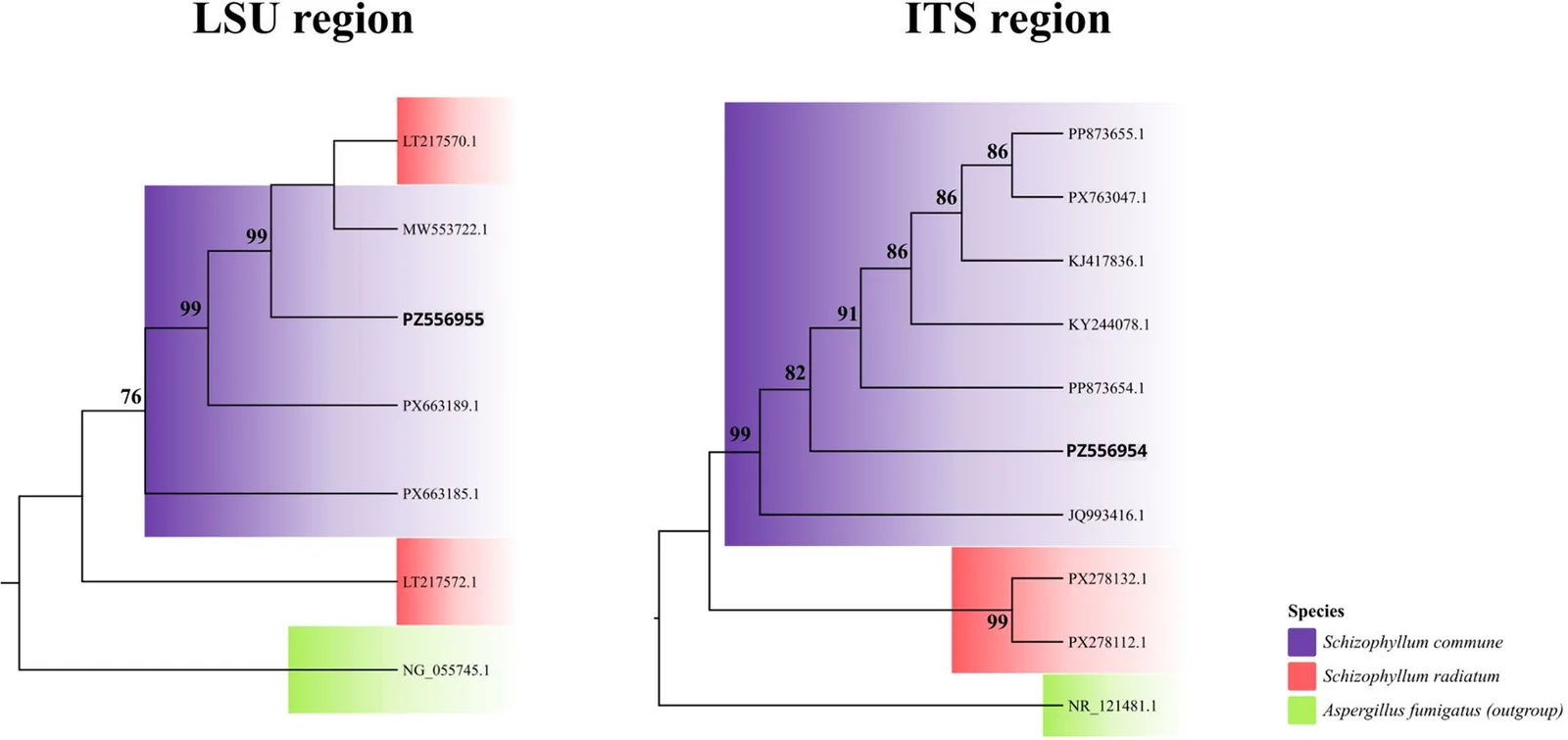
Maximum likelihood phylogenetic trees inferred from internal transcribed spacer (ITS) and nuclear large subunit ribosomal DNA (LSU/NL primers) sequences of *Schizophyllum* spp. Analyses were performed in MEGA12 using the Kimura two-parameter (K2P) substitution model with 1,000 bootstrap replicates. Bootstrap support values (%) are indicated at the nodes. *Aspergillus fumigatus* was used as the outgroup. The isolate obtained in the present study is shown in bold (PZ556954 for ITS and PZ556955 for LSU/NL primers)

Subsequently, BLASTn analysis of the ITS sequence (GenBank accession number PZ556954) revealed exclusively high-scoring matches to *S. commune* sequences deposited in GenBank. Representative isolates, including VXL155 (MW578799.1), RLS40 (PX763053.1), P475 (OP037974.1), INTA-IMC1455 (KY244078.1), URM 8020 (PP873654.1), URM 8772 (PP873655.1), strain 4108 (OR886606.1), and UFMGCB 1509 (KJ417836.1), exhibited 100% nucleotide identity and 100% query coverage. Numerous additional *S. commune* sequences showed nucleotide identities ranging from 99.0% to 99.8%, also with complete query coverage. Importantly, no *S. radiatum* sequences were recovered among ITS sequences. Taken together, the molecular findings support the identification of the fungal agent as *S. commune*. Phylogenetic analysis further supported these findings, as the sequence obtained in this study clustered within the *S. commune* clade with strong bootstrap support (Fig. 4).

Three other harbor seals share the same enclosure and are exposed to the same environmental and dietary conditions; however, none have shown similar clinical abnormalities, and all have remained clinically healthy.

## Discussion

Although *S. commune* infections remain uncommon, human cases have increased in recent decades, likely reflecting advances in diagnostic methods and greater recognition of this fungus as an opportunistic pathogen (Chowdhary et al. 2013; Kleijburg and Wösten 2025). In contrast, veterinary epidemiological data are exceedingly scarce, precluding reliable prevalence estimates and highlighting the significance of the present report.

To the best of our knowledge, this is the first report from South America documenting disseminated *S. commune* infection in a harbor seal (*Phoca vitulina*) maintained under human care. Only two cases of *S. commune* in harbor seals have been reported, both in Japan. Hanafusa et al. (2016) reported a systemic infection associated with pulmonary granuloma formation and fungal encephalitis. The second case described a co-infection with Japanese encephalitis virus (JEV) and *S. commune*, characterized by chronic respiratory disease and extensive pulmonary involvement (Fujii et al. 2024).

Although no specific predisposing factors were identified in this case, chronic stress, associated immunosuppression, and environmental conditions may have contributed to the development of the fungal infection. However, no specific environmental source of exposure or contamination could be identified. In pinnipeds maintained in human care, fungal infections are frequently associated with stress, warm, humid aquatic environments, and impaired immune function (Pollock et al. 2000). In addition, marine mammals face unique immunological challenges, including alterations in mucosal barriers and continuous exposure to moist environments, which may facilitate invasion by opportunistic fungi (Field 2022). In the present case, prior corticosteroid treatment may have further reduced immune resistance, thereby favoring systemic dissemination of the pathogen. These factors may play a role analogous to that described in human cases, in which *S. commune* infects immunocompetent individuals exposed to humid environments or settings contaminated with fungal spores (Itoh et al. 2021; Xue et al. 2025). Notably, three other harbor seals housed in the same enclosure and exposed to the same environmental and dietary conditions remained clinically healthy and showed no similar clinical signs. This observation may suggest that individual host factors also contributed to disease development. Although not specifically investigated in this case, an underlying individual or genetic predisposition associated with impaired immune function cannot be excluded.

The morphologic findings confirm disseminated *S. commune infection*, with involvement of the lungs and central nervous system. The lung lesions corroborate those described in a harbor seal by Hanafusa et al. (2016). Conversely, while Fujii et al. (2024) documented concurrent meningoencephalitis, their neurological findings were largely confounded by a Japanese encephalitis virus co-infection, highlighting the distinct direct fungal neurotropism observed in the present case.

In humans, the pathogenic spectrum of *S. commune* ranges from allergic bronchopulmonary mycosis and chronic rhinosinusitis to necrotizing pneumonia and lung abscesses (Chowdhary et al. 2013). Deep tissue invasion yields local osteolytic lesions, such as hard palate ulcers and sinonasal bone erosion (Sigler et al. 1995), as well as neurotropic dissemination resulting in atypical meningitis, subdural empyema, and cerebral abscesses (Rihs et al. 1996). Peripheral manifestations include subcutaneous granulomas (Tian et al. 2018), otomycosis (Maeda et al. 2019), keratitis, and post-traumatic endophthalmitis (Reddy et al. 2016; Arai et al. 2021). In Brazil, one case of *S. commune* fungemia was described in an HIV-infected patient, demonstrating that the fungus occurs within the country and indicating its potential to cause bloodstream infections with subsequent dissemination to the central nervous system, particularly in severely immunosuppressed individuals. (Oliveira et al. 2017).

The respiratory tract is considered the primary portal of entry for *S. commune*, with infection presumed to occur through inhalation of airborne basidiospores (Chowdhary et al. 2013). *S. commune* is a ubiquitous environmental basidiomycete, commonly associated with decaying wood, plant debris, and other lignocellulosic substrates, and has been isolated from forested areas, urban environments, agricultural settings, and indoor air samples (Sigler et al. 1995; Chowdhary et al. 2013; Mahajan 2022). In the present case, the severe pyogranulomatous pneumonia observed at necropsy suggests that the lungs were the most likely primary site of infection. Pinnipeds possess unique diving adaptations, including repeated exposure to hypoxemia and cyclical alveolar collapse and re-expansion (Kooyman and Ponganis 1998; Ponganis 2015). In other pathogenic fungi, hypoxic microenvironments have been shown to influence fungal physiology and host–pathogen interactions by modulating virulence-associated pathways, lipid metabolism, biofilm formation, and local inflammatory responses (Grahl and Cramer 2010; Grahl et al. 2012). Whether similar mechanisms contribute to the establishment or progression of *S. commune* infection in marine mammals remains unknown.

Infections of the central nervous system caused by fungi such as *Cryptococcus* spp., *Aspergillus* spp., and members of the order Mucorales may occur following hematogenous dissemination and subsequent penetration of the blood–brain barrier (Reyes and Shinohara 2022). The intravascular fungal hyphae observed in the present case are consistent with hematogenous dissemination, although they do not establish the route by which the fungus reached the central nervous system. In humans and dogs, retrograde invasion through the olfactory pathway across the cribriform plate has also been described in cryptococcal infections (Sabiiti and May 2014). Once established within the central nervous system, fungal pathogens may benefit from the distinctive immunometabolic characteristics of neural tissue, including its high lipid content and specialized immune environment (Reyes and Shinohara 2022). Although central nervous system involvement has been reported in both humans and dogs infected with *S. commune* (Rihs et al. 1996; Nagashima et al. 2024), the mechanisms underlying neuroinvasion by this fungus remain unknown.

Sequencing of the D1/D2 region enabled identification of the fungal isolate at the genus level but did not reliably discriminate among closely related *Schizophyllum* species due to their high sequence conservation (Kurtzman and Robnett 1997). In contrast, ITS sequencing followed by BLASTn and phylogenetic analyses provided sufficient taxonomic resolution to unequivocally identify the isolate as *S. commune*, distinguishing it from *S. radiatum* (Schoch et al. 2012; Siqueira et al. 2016).

Overall, this report contributes to the limited knowledge of the pathogenic potential of *S. commune* in aquatic mammals and emphasizes the clinical relevance of opportunistic fungal infections in pinnipeds under human care. The concurrence of progressive clinical manifestations, extensive anatomopathological alterations, and definitive etiological confirmation by molecular techniques supports including *S. commune* in the differential diagnosis of granulomatous pneumonia and encephalitis in this species. Moreover, the paucity of available data highlights the need for further studies to clarify predisposing factors, routes of dissemination, and preventive measures, particularly in captive environments where host immunity and environmental conditions may predispose to infection establishment and progression.

## Supplementary Information

Below is the link to the electronic supplementary material.

Supplementary Material 1 (DOCX 19.2 KB)

Supplementary Material 2 (DOCX 16.7 KB)

## Data Availability

The sequence data generated during this study are available in GenBank under accession numbers PZ556954 and PZ556955. All other data generated or analyzed during this study are included in this published article.

## Declarations

### Generative AI and AI-assisted technologies

During the preparation of this work, the author(s) used ChatGPT (version 5.5) in order to improve the grammar, readability, and English language styling of the manuscript. This tool was used in compliance with the publisher's integrity guidelines and the Brazilian National Council for Scientific and Technological Development (CNPq No. 2.664/2026). After using this tool, the author(s) thoroughly reviewed, verified, and edited the content as needed. The author(s) take(s) full responsibility and accountability for the final text and the scientific integrity of the publication.

## References

[CR1] Arai Y, Hashimoto Y, Makino S (2021) *Schizophyllum commune*-induced postoperative endophthalmitis. QJM 114:517–518. 10.1093/qjmed/hcab047 33682873

[CR2] Chowdhary A, Randhawa HS, Gaur SN et al (2013) *Schizophyllum commune* as an emerging fungal pathogen: a review and report of two cases. Mycoses 56:1–10. 10.1111/j.1439-0507.2012.02190.x 22524529

[CR3] Ehlers B, Dural G, Yasmum N et al (2008) Novel mammalian herpesviruses and lineages within the Gammaherpesvirinae: cospeciation and interspecies transfer. J Virol. 10.1128/JVI.02646-07 PMC2268488 18216123

[CR4] Field CL (2022) Mycotic diseases of marine mammals. In: Aiello SE, Moses MA (eds) MSD veterinary manual, 12th edn. Merck & Co., Inc., Kenilworth, pp 230–257

[CR5] Fujii M, Ito S, Katsumata E et al (2024) Japanese encephalitis virus and *Schizophyllum commune* co-infection in a harbor seal in Japan. Vet Sci 11:215. 10.3390/vetsci11050215 PMC11125775 38787188

[CR6] Go H, Yeon KD, Lee JH et al (2022) Disseminated fungal infection with *Aspergillus versicolor* and *Schizophyllum commune* in a dog. Med Mycol Case Rep 38:25–29. 10.1016/j.mmcr.2022.09.005 PMC9561684 36245568

[CR7] Grahl N, Cramer RA (2010) Regulation of hypoxia adaptation: an overlooked virulence attribute of pathogenic fungi? Med Mycol 48:1–15. 10.3109/13693780902947342 PMC2898717 19462332

[CR8] Grahl N, Shepardson KM, Chung D, Cramer RA (2012) Hypoxia and fungal pathogenesis: to air or not to air? Eukaryot Cell 11:560–570. 10.1128/EC.00031-12 PMC3346435 22447924

[CR9] Hakamata M, Kano R, Kondo H, Watari T (2019) Canine fungal osteomyelitis. Mycopathologia 184:707–708. 10.1007/s11046-019-00362-7 31346858

[CR10] Hanafusa Y, Hirano Y, Watabe H et al (2016) First isolation of *Schizophyllum commune* in a harbor seal (*Phoca vitulina*). Med Mycol 54:492–499. 10.1093/mmy/myw008 26933208

[CR11] Itoh N, Akazawa N, Murakami H et al (2021) A *Schizophyllum commune* fungus ball in a lung cancer cavity: a case report. BMC Infect Dis 21:1052. 10.1186/s12879-021-06739-8 PMC8502358 34627188

[CR12] Kano R, Oomae S, Nakano Y et al (2002) First report on *Schizophyllum commune* from a dog. J Clin Microbiol 40:3535–3537. 10.1128/JCM.40.9.3535-3537.2002 PMC130828 12202614

[CR13] Kleijburg FEL, Wösten HAB (2025) The versatility of *Schizophyllum commune* in nature and application. Fungal Biol Rev 53:100431. 10.1016/j.fbr.2025.100431

[CR14] Kooyman GL, Ponganis PJ (1998) The physiological basis of diving to depth: birds and mammals. Annu Rev Physiol 60:19–32. 10.1146/annurev.physiol.60.1.19 9558452

[CR15] Kurtzman CP, Robnett CJ (1997) Identification of clinically important ascomycetous yeasts based on nucleotide divergence in the 5’ end of the large-subunit (26S) ribosomal DNA gene. J Clin Microbiol 35:1216–1223. 10.1128/jcm.35.5.1216-1223.1997 PMC232732 9114410

[CR16] Leaw SN, Chang HC, Sun HF et al (2006) Identification of medically important yeast species by sequence analysis of the internal transcribed spacer regions. J Clin Microbiol 44:693–699. 10.1128/JCM.44.3.693-699.2006 PMC1393093 16517841

[CR17] Maeda M, Maeda T, Nakamura A, Komatsu M (2019) A case of otitis externa caused by *Schizophyllum commune*: an approach to antimicrobial stewardship using Gram staining of otorrhea in a medical clinic. J Infect Chemother 25:731–734. 10.1016/j.jiac.2019.03.005 30905630

[CR18] Mahajan M (2022) *Schizophyllum commune*. Emerg Infect Dis 28:725. 10.3201/eid2803.211051

[CR19] Mori T, Seki A, Kano R et al (2009) Mycotic osteomyelitis caused by *Schizophyllum commune* in a dog. Vet Rec 165:350–351. 10.1136/vr.165.12.350 19767641

[CR20] Nagashima T, Tsumoto S, Yazawa D et al (2024) Disseminated granulomatous encephalitis caused by *Schizophyllum commune* in a dog with severe neurological signs. J Comp Pathol 213:73–77. 10.1016/j.jcpa.2024.07.004 39146622

[CR21] Noguchi S, Toyota K, Ozaki M et al (2023) Successful management of nasopharyngitis caused by *Schizophyllum commune* in a captive cheetah (*Acinonyx jubatus*). J Vet Med Sci 85:1225–1229. 10.1292/jvms.23-0273 PMC10600532 37574282

[CR22] Oliveira MME, Lemos AS, Gonçalves MLC et al (2017) Fungemia associated with *Schizophyllum commune* in Brazil. PLoS Negl Trop Dis 11:e0005549. 10.1371/journal.pntd.0005549 PMC5490937 28662024

[CR23] Pollock CG, Rohrbach B, Ramsay EC (2000) Fungal dermatitis in captive pinnipeds. J Zoo Wildl Med 31:374–378. 10.1638/1042-7260(2000)031[0374:fdicp]2.0.co;2 11237146

[CR24] Ponganis PJ (2015) Diving physiology of marine mammals and seabirds. Cambridge University Press, Cambridge

[CR25] Reddy AK, Ashok R, Majety M et al (2016) Fungal keratitis due to *Schizophyllum commune*: an emerging pathogenic fungus. Mycoses 59:757–759. 10.1111/myc.12527 27402206

[CR26] Reyes EY, Shinohara ML (2022) Host immune responses in the central nervous system during fungal infections. Immunol Rev 311:50–74. 10.1111/imr.13101 PMC9489659 35672656

[CR27] Rihs JD, Padhye AA, Good CB (1996) Brain abscess caused by *Schizophyllum commune*: an emerging basidiomycete pathogen. J Clin Microbiol 34:1628–1632. 10.1128/jcm.34.7.1628-1632.1996 PMC229083 8784558

[CR28] Rojas M, González I, Pavón MÁ et al (2010) Novel TaqMan real-time polymerase chain reaction assay for verifying the authenticity of meat and commercial meat products from game birds. Food Addit Contam Part A 27:749–763. 10.1080/19440040903503070 20169483

[CR29] Sabiiti W, May RC (2014) Pathogens penetrating the central nervous system: infection pathways and the cellular and molecular mechanisms of invasion. Clin Microbiol Rev 27:691–726. 10.1128/cmr.00118-13 PMC4187632 25278572

[CR30] Schoch CL, Seifert KA, Huhndorf S et al (2012) Nuclear ribosomal internal transcribed spacer (ITS) region as a universal DNA barcode marker for Fungi. Proc Natl Acad Sci USA 109:6241–6246. 10.1073/pnas.1117018109 PMC3341068 22454494

[CR31] Sigler L, de la Maza LM, Tan G et al (1995) Diagnostic difficulties caused by a nonclamped *Schizophyllum commune* isolate in a case of fungus ball of the lung. J Clin Microbiol 33:1979–1983. 10.1128/jcm.33.8.1979-1983.1995 PMC228320 7559933

[CR32] Siqueira JPZ, Sutton D, Gené J et al (2016) *Schizophyllum radiatum*, an emerging fungus from human respiratory tract. J Clin Microbiol 54:2491–2497. 10.1128/JCM.01170-16 PMC5035413 27440814

[CR33] Tamura K, Stecher G, Kumar S (2021) MEGA11: Molecular Evolutionary Genetics Analysis version 11. Mol Biol Evol 38:3022–3027. 10.1093/molbev/msab120 PMC8233496 33892491

[CR34] Tanaka H, Takizawa K, Baba O et al (2008) Basidiomycosis: *Schizophyllum commune* osteomyelitis in a dog. J Vet Med Sci 70:1257–1259. 10.1292/jvms.70.1257 19057147

[CR35] Tian L, Mu Y, Zhang H et al (2018) First report on cutaneous infectious granuloma caused by *Schizophyllum commune*. BMC Infect Dis 18:286. 10.1186/s12879-018-3187-5 PMC6019233 29940940

[CR36] VanDevanter DR, Warrener P, Bennett L et al (1996) Detection and analysis of diverse herpesviral species by consensus primer PCR. J Clin Microbiol 34:1666–1671. 10.1128/jcm.34.7.1666-1671.1996 PMC229091 8784566

[CR37] Won EJ, Shin JH, Lim SC et al (2012) Molecular identification of *Schizophyllum commune* as a cause of allergic fungal sinusitis. Ann Lab Med 32:375–379. 10.3343/alm.2012.32.5.375 PMC3427828 22950076

[CR38] Xue C, Shen J, Song J et al (2025) Pulmonary infection caused by *Schizophyllum commune*: a case study. Front Med (Lausanne) 12:1475896. 10.3389/fmed.2025.1475896 PMC12206706 40589965

[CR39] Yoshizawa M, Kawarai S, Torii Y et al (2017) Eosinophilic plasmacytic conjunctivitis concurrent with gingival fistula caused by *Schizophyllum commune* in a captive cheetah (*Acinonyx jubatus*). Med Mycol Case Rep 18:34–39. 10.1016/j.mmcr.2017.09.001 PMC5633821 29034160

