# Supplementary material for: Disseminated and fatal infection by Schizophyllum commune in a harbor seal (Phoca vitulina) kept under human care in Brazil
Source: Vet Res Commun. 2026 Oct 7;50(6):616. doi: 10.1007/s11259-026-11559-9 (PMC13642384; doi:10.1007/s11259-026-11559-9)
Supplement: Supplementary file 1 [file 11259_2026_11559_MOESM1_ESM.docx]

**Supplementary Table 1.** Hematologic and serum biochemical findings.

| Parameter | 10 Mar 2022 | 06 Jan 2023 | 11 Jan 2023 | 14 Jan 2023 | Reference interval (ISIS, 2002) |
| --- | --- | --- | --- | --- | --- |
| RBC (×10⁶/µL) | 4.74 | 1.96 | 3.50 | 3.27 | 4.13–5.49 |
| Hemoglobin (g/dL) | 21.1 | 10.2 | 13.5 | 12.5 | 18.0–26.6 |
| Hematocrit (%) | 55.7 | 23 | 37 | 35 | 48.4–59.6 |
| MCV (fL) | 117.5 | 117.35 | 105.71 | 107.03 | 97.5–133 |
| MCH (pg) | 44.5 | 52.04 | 38.57 | 38.23 | 35.1–63.7 |
| MCHC (g/dL) | 37.9 | 44.35 | 36.49 | 35.71 | 33–47 |
| Total leukocytes (/µL) | 23,600 | 10,925 | 34,860 | 36,802 | 5,778–13,404 |
| Neutrophils (/µL) | 18,880 | 8,631 | 28,237 | 32,754 | 3,236–9,798 |
| Eosinophils (/µL) | 708 | 874 | 3,486 | 736 | 70–608 |
| Lymphocytes (/µL) | 2,360 | 1,093 | 1,046 | 1,840 | 1,219–2,925 |
| Monocytes (/µL) | 1,652 | 328 | 2,092 | 1,472 | 114–1,356 |
| Basophils (/µL) | 0 | 0 | 0 | 0 | 51–361 |
| Platelets (/µL) | 340,000 | ND | 598,000 | 411,000 | 212,000–474,000 |
| Total protein (g/dL) | 8.10 | 5.9 | 8.6 | 8.0 | 6.8–8.4 |
| Urea (mg/dL) | 92 | 53 | 103 | 154 | 31–47 |
| Creatinine (mg/dL) | 0.80 | 1.5 | 1.3 | NR | 1.0–1.8 |
| ALT (U/L) | 31 | 95 | 79 | 68 | 18–64 |
| AST (U/L) | 83 | NR | 235 | 136 | 24–84 |
| GGT (U/L) | 36 | NR | 34 | 25 | 2–12 |
| ALP (U/L) | 5 | NR | 19 | 21 | 18–72 |
| Albumin (g/dL) | 4.10 | 3.52 | 3.7 | 3.5 | 3.5–4.3 |
| Globulins (g/dL) | 4.00 | 2.38 | 4.9 | 4.5 | 3.1–4.5 |
| Fibrinogen (g/dL) | 600 | NR | 400 | 400 | 164 |
| Glucose (mg/dL) | 134.4 | NR | NR | 94 | 188 – 164 |
| Creatine kinase (U/L) | 1,377 | NR | NR | NR | NA* |
| Lactate dehydrogenase (U/L) | NR | NR | 680 | 1,991 | 130 – 692 |
| Phosphorus (mg/dL) | 5.80 | NR | 7.6 | 6.5 | 3,2 – 6,4 |
| Sodium (mmol/L) | 150.0 | NR | NR | NR | 148–159 |
| Potassium (mmol/L) | 3.90 | NR | NR | NR | 3.5–5.2 |
| Chloride (mmol/L) | 105.0 | NR | NR | NR | 103–117 |
| Total calcium (mg/dL) | 8.9 | NR | 6.92 | NR | 8.3-10.1 |
| Blood culture | NR | NR | Negative | NR | Negative |

Abbreviations: NR, not reported; ND, not determined due to sample clotting. Reference intervals obtained from ISIS (2002) when available in the laboratory reports. Neutrophil hypersegmentation was reported on 11 Jan, 14 Jan and 10 Mar 2022; cytoplasmic basophilia and dense granulation were reported on 11 Jan 2023.

References values: Hall AJ, Russell DJF, Thompson PM, Milne R, Moss SEW, Armstrong HC, Kershaw JL. **Clinical Chemistry Reference Intervals for Health Assessment in Wild Adult Harbour Seals.** *Animals*. 2025;15:3429; International Species Information System (ISIS). **Physiological Data Reference Values.** Apple Valley, MN: International Species Information System; 2002. NA*:, reference interval not available for *Phoca vitulina.*
