# Supplementary material for: Disseminated and fatal infection by Schizophyllum commune in a harbor seal (Phoca vitulina) kept under human care in Brazil
Source: Vet Res Commun. 2026 Oct 7;50(6):616. doi: 10.1007/s11259-026-11559-9 (PMC13642384; doi:10.1007/s11259-026-11559-9)
Supplement: Supplementary file 2 [file 11259_2026_11559_MOESM2_ESM.docx]

**Supplementary Table 2.** Results of the molecular and serological diagnostic panel performed during the clinical investigation in March 2022.

| **Diagnostic method** | **Sample** | **Target pathogen** | **Results** |
| --- | --- | --- | --- |
| PCR | Ocular and nasal swabs | Influenza vírus | Negative |
| PCR | Ocular and nasal swabs | Herpesvirus | Negative |
| PCR | Ocular and nasal swabs | Adenovirus | Negative |
| PCR | Ocular and nasal swabs  Blood | Morbilivirus | Negative |
| PCR | Ocular and nasal swabs | Canine Adenovirus 2 | Negative |
| PCR | Ocular and nasal swabs | Canine coronavirus | Negative |
| PCR | Ocular and nasal swabs | Canine parainfluenza vírus | Negative |
| PCR | Ocular and nasal swabs  Blood | West Nile virus | Negative |
| PCR | Ocular and nasal swabs | *Bordetella bronchiseptica* | Negative |
| PCR | Ocular and nasal swabs | *Mycoplasma cynos* | Negative |
| PCR | Ocular and nasal swabs | *Streptococcus equi* subsp. *zooepidemicus* | Negative |
| PCR | Ocular and nasal swabs  Blood | *Bartonella* sp. | Negative |
| PCR | Ocular and nasal swabs | *Borrelia burgdorferi* | Negative |
| PCR | Ocular and nasal swabs  Blood | *Neospora caninum* | Negative |
| PCR | Ocular and nasal swabs | Sarcocystidae | Negative |
| PCR | Ocular and nasal swabs  Blood | *Blastomyces dermatitidis* | Negative |
| PCR | Ocular and nasal swabs  Blood | *Coccidioides* sp. | Negative |
| PCR | Ocular and nasal swabs  Blood | *Cryptococcus* sp. | Negative |
| PCR | Ocular and nasal swabs  Blood | *Histoplasma capsulatum* | Negative |
| PCR | Ocular and nasal swabs  Blood | *Toxoplasma gondii* | Negative |
| Hemagglutination assay | Blood | *Toxoplasma gondii* | IgM: Negative; IgG: Negative |

Abbreviations: PCR, polymerase chain reaction.
